# The interaction between osteosarcoma and other cells in the bone microenvironment: From mechanism to clinical applications

**DOI:** 10.3389/fcell.2023.1123065

**Published:** 2023-05-03

**Authors:** Jin Zeng, Yi Peng, Dong Wang, Khan Ayesha, Shijie Chen

**Affiliations:** ^1^ Department of Spine Surgery, The Third Xiangya Hospital of Central South University, Changsha, China; ^2^ Xiangya School of Medicine, Central South University, Changsha, China; ^3^ Shanghai Key Laboratory of Regulatory Biology, Institute of Biomedical Sciences and School of Life Sciences, East China Normal University, Shanghai, China

**Keywords:** osteosarcoma, bone microenvironment, cell cross-talk, mechanism, clinical applications

## Abstract

Osteosarcoma is a primary bone tumor with a high mortality rate. The event-free survival rate has not improved significantly in the past 30 years, which brings a heavy burden to patients and society. The high heterogeneity of osteosarcoma leads to the lack of specific targets and poor therapeutic effect. Tumor microenvironment is the focus of current research, and osteosarcoma is closely related to bone microenvironment. Many soluble factors and extracellular matrix secreted by many cells in the bone microenvironment have been shown to affect the occurrence, proliferation, invasion and metastasis of osteosarcoma through a variety of signaling pathways. Therefore, targeting other cells in the bone microenvironment may improve the prognosis of osteosarcoma. The mechanism by which osteosarcoma interacts with other cells in the bone microenvironment has been extensively investigated, but currently developed drugs targeting the bone microenvironment have poor efficacy. Therefore, we review the regulatory effects of major cells and physical and chemical properties in the bone microenvironment on osteosarcoma, focusing on their complex interactions, potential therapeutic strategies and clinical applications, to deepen our understanding of osteosarcoma and the bone microenvironment and provide reference for future treatment. Targeting other cells in the bone microenvironment may provide potential targets for the development of clinical drugs for osteosarcoma and may improve the prognosis of osteosarcoma.

## 1 Introduction

Osteosarcoma (OS) is the most common primary malignant bone tumor, with most cases occurring in children and young adults between the ages of 10 and 30 years. The most common sites of tumor formation are those with the most extensive longitudinal bone growth: the knee (distal femur and proximal tibia) and the shoulder (proximal humerus) (Meltzer and Helman, 2021). The main clinical manifestations of OS are bone pain, swelling, and dysfunction. The onset of OS is often hidden and difficult to detect early. OS is prone to distant hematogenous metastasis, especially to the lung. Almost all patients are considered to have subclinical small metastatic disease at the time of diagnosis, and only 15%–20% of these patients are successfully detected to have metastasis (Sheng et al., 2021). At present, the treatment of OS is based on its classification and staging, which is the preferred surgery for both low-grade and high-grade OS. Surgery combined with preoperative and post-operative chemotherapy was selected for high-grade OS, while surgery and other adjuvant chemoradiotherapy were selected for low-grade OS (Grimer, 2005; Harrison et al., 2018). The first-line chemotherapy regimen consisted of doxorubicin, cisplatin, and high-dose methotrexate, and the second-line chemotherapy regimen consisted of ifosfamide, cyclophosphamide, etoposide, carboplatin, gemcitabine, docetaxel, sorafenib, rigofenib, and samarium. Ectodyl tripeptide has been approved in Europe for the treatment of postoperative OS in patients under 30 years of age (Zhu et al., 2022). Despite long exploration, 5-year event-free survival in patients with OS has not improved significantly over the past few decades. Therefore, the treatment of OS still needs further exploration.

**FIGURE 1:**
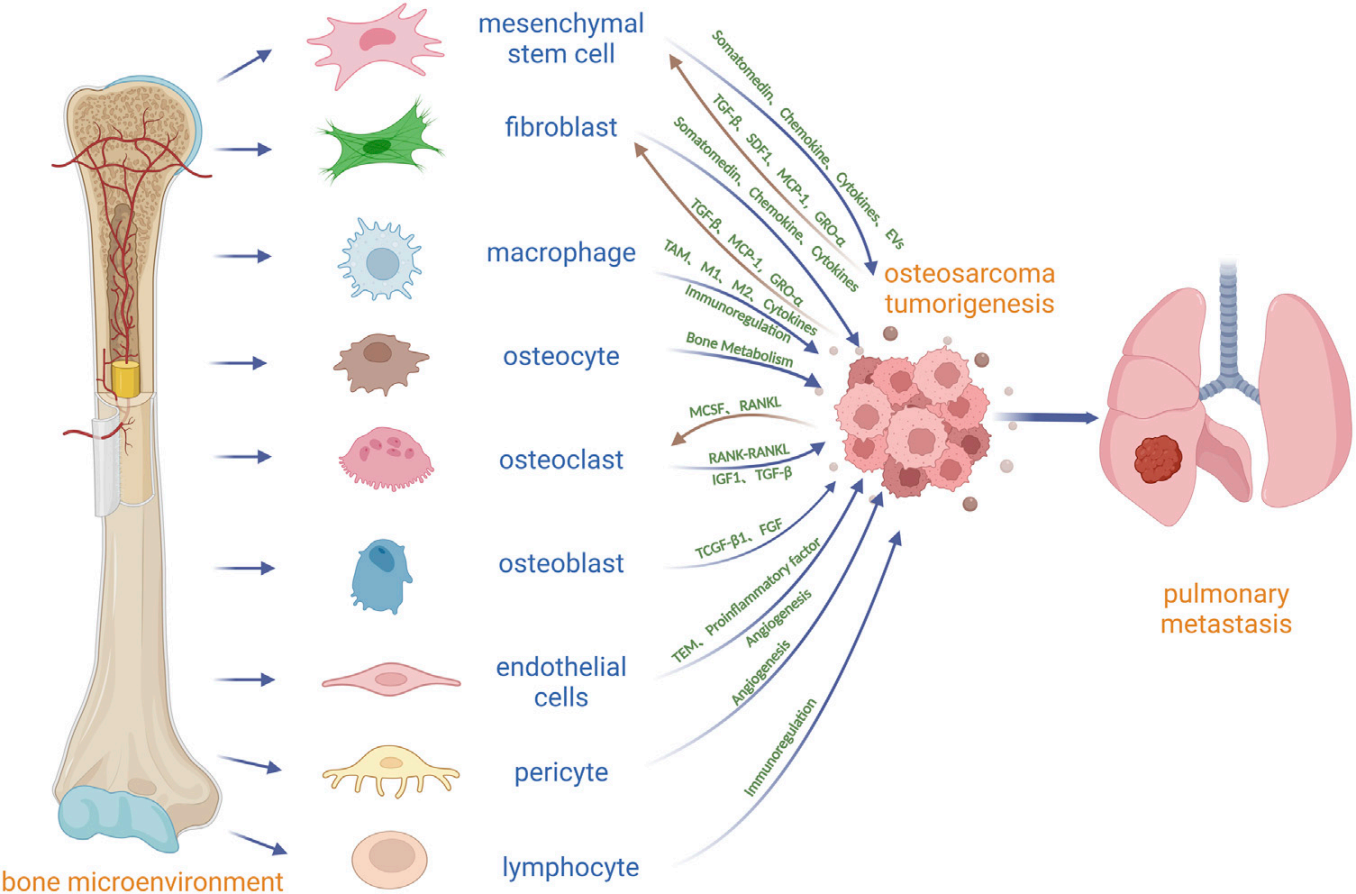
Diagrammatic drawing of the cross-talk between OS and other cells in the bone microenvironment. MSC and fibroblast can secrete some factors or choose the extracellular vesicles as the carrier to transport somatomedin, chemokine and Cytokines which promote OS growth and metastasis. In addition, OS can induce the migration of MSC into OS by TGF-β and matrix derived factor (SDF1), and can induce the differentiation of MSC into cancer-associated fibroblasts (CAF) by CP-1, GRO-α and TGF-β. Macrophages can promote OS growth, invasion and metastasis by differentiating into TAM, changing the ratio of M1 to M2 macrophages, secreting cytokines and immune regulation. Osteocytes can directly participate in the formation of osteolytic and osteoblastic lesions by regulating osteoclasts and osteoblasts in OS. It is believed that OS stimulates osteoclast differentiation and maturation through secretion of M-CSF and RANKL, and differentiated and mature osteoclasts further stimulate OS growth through bone resorption, such as the release of IGF1 or TGF-β in bone matrix. Osteoblasts may be related to the origin of OS through TGF-β1 and fibroblast growth factor (FGF). Vascular endothelial cells may promote the proliferation and metastasis of OS by promoting the transendothelial migration (TEM) of OS, secreting proinflammatory factors and promoting angiogenesis. Pericytes may be important mediators of OS angiogenesis. Lymphocytes communicate with OS through immune regulation.

At present, the focus on tumor has been extended from the tumor cell itself to the tumor microenvironment (TME), which can promote tumor cell proliferation, metastasis, anti-apoptosis and drug resistance (Zhu et al., 2022). OS is located in the bone microenvironment, which is a very special and complex and highly dynamic environment, by bone cells (osteoclasts, osteoblasts, osteocytes), stromal cells (between mesenchymal stem cells, fibroblasts), blood vessel cells (endothelial cells and pericytes), immune cells (macrophages, lymphocytes) and mineralization of extracellular matrix (Corre et al., 2020) (Figure 1). Under physiological conditions, skeletal, vascular, and stromal cells maintain bone homeostasis through paracrine and cellular communication, and tumor cells can manage to master skeletal physiological pathways to their advantage in this microenvironment for survival and growth. There are many environmental signals involved between OS and the bone microenvironment, which are induced by a variety of cytokines, chemokines and soluble growth factors (Alfranca et al., 2015). In the bone microenvironment, osteoclasts can promote the growth of OS by releasing insulin-like growth factor 1 (IGF1) and transforming growth factor β (TGF-β) from the bone matrix (Norregaard et al., 2021) (Figure 1; Table 1). Osteoblasts may be the precursor cells of OS. Mesenchymal stem cell (MSC) can secrete a range of cytokines, and extracellular vesicles and differentiate into cancer-associated fibroblasts to directly promote OS growth and metastasis (Corre et al., 2020) (Figure 1). Vascular endothelial cells and pericytes may promote OS growth and metastasis by regulating angiogenesis (Figure 1). Macrophages may contribute to OS growth by promoting angiogenesis, immunosuppression, and chronic inflammation (Figure 1). The role of osteocytes and fibroblasts in OS is still unclear. Osteocytes may participate in the growth of OS by regulating bone balance, and fibroblasts may promote the growth and metastasis of OS by differentiating into cancer-related fibroblasts to secrete cytokines and extracellular vesicles (Figure 1). Lymphocytes may be involved in the growth and metastasis of OS through immunosuppression and evasion (Figure 1). Current protocols do not eradicate all OS cells in the body, especially metastatic and circulating OS cells, which may lead to recurrence and metastasis. Targeting other cells in the bone microenvironment is expected to inhibit the growth of OS and improve 5-year event-free survival in patients with OS. Drugs targeting the TME will have potential applications.

**TABLE 1:** Chemokines and other factors from cells in the bone microenvironment that affect OS.

Factor	Source	Pathway	Target	*In Vitro* or *In Vivo*	References
IGF1	Osteoclast	IGF-1/IGF-1R in OS	OS	*In Vitro*	Sergi et al. (2019), Dodington et al. (2021)
	Cancer-associated fibroblast	IGF-1/IGF-1R/PI3K/AKT in OS	OS	*In Vitro*	Sergi et al. (2019)
TGF-β	Osteoclast	TGF-β/Smad3 in OS	OS	*In Vitro*	Saito et al. (2018), Chang et al. (2021)
	OS extracellular vesicle	IL-6/STAT3 signaling pathway in MSC	MSC	*In Vitro* and *in Vivo*	Pietrovito et al. (2018)
	Cancer-associated fibroblast	PI3K/AKT signaling pathway	OS	*In Vitro*	Lamora et al. (2016), Ma et al. (2022)
	Vascular endothelial cell	TGF-β/TGF-β R in Vascular pericyte	Vascular pericyte	*In Vitro*	Zonneville et al. (2018)
MCSF(CSF1)	OS	MCSF/CSF1R in Osteoclast	Osteoclast	*In Vitro*	Ross and Teitelbaum (2005), Gyori and Mocsai (2020)
	Cancer-associated fibroblast	CSF1/CSF1R in OS	OS	*In Vitro* and *in Vivo*	Smeester et al. (2020)
RANKL	OS	RANK/RANKL in Osteoclast	Osteoclast	*In Vitro*	Navet et al. (2018), Rao et al. (2018)
RANK	Osteoclast	RANK/RANKL in OS	OS	*In Vitro*	Navet et al. (2018), Rao et al. (2018)
SDF1	OS	Trans-differentiate into cancer-associated fibroblasts in MSC	MSC	*In Vitro*	Pietrovito et al. (2018)
	MSC extracellular vesicle	SDF-1/CXCR4 in OS	OS	*In Vitro*	Wei et al. (2022)
MCP-1	OS	MAT in MSC	MSC	*In Vitro*	Yang et al. (2020)
GRO-α(CXCL1)	OS	Transdifferentiate into cancer-associated fibroblasts in MSC	MSC	*In Vitro*	Yang et al. (2020)
	MSC	NF-κB signaling pathway in OS	OS	*In Vitro*	Yang et al. (2020)
IL–8	MSC	NF-κB signaling pathway in OS	OS	*In Vitro*	Yang et al. (2020)
	MSC and OS	IL-8/CXCR1/Akt signaling pathway, MAT in OS	OS	*In Vitro* and *in Vivo*	Yang et al. (2020)
	MSCs, Cancer-associated fibroblast	MAT in OS	OS	*In Vitro*	Yang et al. (2020)
CSF2/GMCSF	MSC	NF-κB signaling pathway in OS	OS	*In Vitro*	Yang et al. (2020)
CSF3/GCSF	MSC	NF-κB signaling pathway in OS	OS	*In Vitro*	Yang et al. (2020)
BMP2	MSC	NF-κB signaling pathway in OS	OS	*In Vitro*	Yang et al. (2020)
CCL5	MSC	NF-κB signaling pathway in OS	OS	*In Vitro*	Yang et al. (2020)
	Cancer-associated fibroblast	NF-κB signaling pathway in OS	OS	*In Vitro*	Kalluri (2016b), Avnet et al. (2017)
CXCL5	MSC	NF-κB signaling pathway in OS	OS	*In Vitro*	Yang et al. (2020)
	Cancer-associated fibroblast	NF-κB signaling pathway in OS	OS	*In Vitro*	Dang et al. (2017)
IL-6	MSC	IL-6/STAT3 signaling pathway in OS	OS	*In Vitro* and *in Vivo*	Yang et al. (2020)
	MSC	NF-κB signaling pathway in OS	OS	*In Vitro*	Yang et al. (2020)
	Cancer-associated fibroblast	MAT in OS	OS	*In Vitro*	Yang et al. (2020)
Has-lncRNA MALAT1	MSC	lncRNA MALAT1/miR-143/NRSN2/Wnt/β-Catenin signaling pathway in OS	OS	*in Vitro* and *in Vivo*	Li et al. (2021)
Has- lncRNA PVT1	MSC	PVT1/ERG signaling pathway in OS	OS	*In Vitro*	Zhao et al. (2019)
Has-miR-150	MSC	IGF2BP1 in OS	OS	*In Vitro*	Xu et al. (2020)
Has-miR-206	MSC	TRA2B in OS	OS	*In Vitro*	Zhang et al. (2020)
CXCL9	Cancer-associated fibroblast	CXCL9/CXCR3 in OS	OS	*In Vitro*	Yu et al. (2020)
CXCL10	Cancer-associated fibroblast	CXCL9/CXCR3 in OS	OS	*In Vitro*	Niu et al. (2020)
CXCL12	Cancer-associated fibroblast	CXCL9/CXCR3 in OS	OS	*In Vitro*	Li et al. (2018)
HGF	Cancer-associated fibroblast	HGF/c-Met signaling pathway in OS	OS	*In Vitro*	Wen et al. (2020)
CTGF	Cancer-associated fibroblast	MCM8/CTGF signaling pathway in OS	OS	*in Vitro* and *in Vivo*	Ren et al. (2021)
PDGF	Cancer-associated fibroblast	PDGF/PDGFRβ in OS	OS	*In Vitro*	Xing et al. (2020)
VEGF	Cancer-associated fibroblast	VEGF/VEGFR in OS	OS	*In Vitro*	Chellini et al. (2018)
IL-1	Cancer-associated fibroblast	IL-1/IL-1R in OS	OS	*In Vitro*	Yati et al. (2022)
IL-4	Cancer-associated fibroblast	IL-4/IL-4R in Macrophage	OS	*In Vitro*	Deng et al. (2020)
IL-10	Cancer-associated fibroblast	NF-κB signaling pathway in OS	OS	*In Vitro*	Raucci et al. (2019)
LIF	Cancer-associated fibroblast	NOTCH1 signaling pathway in OS	OS	*In Vitro*	Lu et al. (2020b)
PGE2	Cancer-associated fibroblast	NF-κB/COX-2 signaling pathway in OS	OS	*In Vitro*	Sun et al. (2021)
VEGF-A	Vascular endothelial cell	VEGF-A/Spred-1	Vascular	*In Vitro*	Wang et al. (2008)
PDGF-B	Vascular endothelial cell	PDGF-B/PDGFRβ in vascular pericyte	Vascular pericyte	*In Vitro*	Lindblom et al. (2003)
FAK	Vascular pericyte	Gas6/Axl signaling pathway in melanoma	Melanoma	*in Vitro* and *in Vivo*	Lechertier et al. (2020)
CCL18	Macrophage	COX-2/STAT3signaling pathway in OS	OS	*in Vitro* and *in Vivo*	Han et al. (2019)

In OS, research and development of new treatments mainly face two difficulties: first, the height of OS cells heterogeneity leads to there being no specific therapeutic targets; secondly, the bone microenvironment composed of various active cells, through a variety of soluble factors and extracellular matrix are interconnected and intensive communication, make the inefficiency of the current treatment of OS. In this review, we describe the complex interactions of osteoclasts, osteoblasts, osteocytes, mesenchymal stem cells, fibroblasts, endothelial cells, pericytes, macrophages, lymphocytes, and OS cells in the OS microenvironment, as well as potential therapeutic strategies and clinical applications, linking the treatment of OS to cellular interactions between cells in the bone microenvironment. The ultimate goal is to provide more information and insight for understanding and treating OS.

## 2 Osteoclast

**FIGURE 2:**
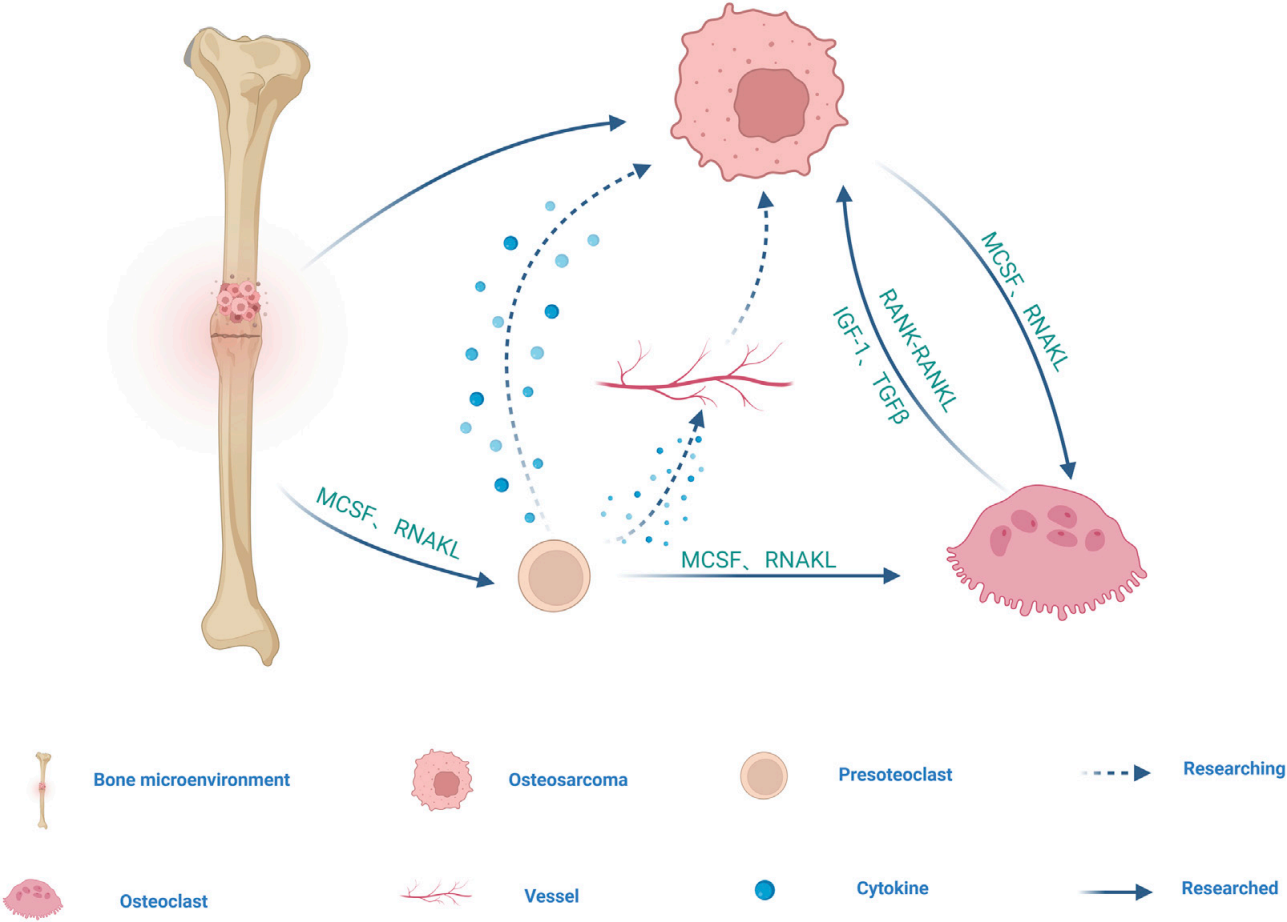
Schematic diagram of the cell cross-talk between OS and OC cell line. Mononuclear macrophages derived from bone marrow differentiate into POC induced by receptors for MCSF and RANKL, and subsequently multiple POC fuse into OC in response to MCSF and RANKL. POC may promote OS growth and metastasis by secreting cytokines and regulating angiogenesis. MCSF and RANKL produced by OS directly and indirectly stimulate OC differentiation and maturation, and differentiated and mature OC further stimulate OS growth by releasing growth factors and minerals during bone resorption, such as IGF1 or TGF-β. RANK-containing exosomes secreted by OC may act on RANKL expressed on OS cells to promote OS progression through RANK-RANKL reverse signaling. The arrow represents the direction, the dotted line represents the unproven, and the solid line represents the proven.

Osteoclasts (OC) originates from hematopoietic stem cells (HSCS in the bone marrow and are differentiated by monocyte/macrophage colony-stimulating factor (M-CSF) and receptor for NF-κB ligand (RANKL) (Figure 2). M-CSF mainly promotes the proliferation and survival of preosteoclast (POC). RANKL is the main factor driving the differentiation of OC precursors into OC (Feng and Teitelbaum, 2013). OC exist only in bone and play a key role in bone resorption and bone remodeling as bone resorptive cells, participating in the pathogenesis of various bone diseases (Ono and Nakashima, 2018).

OS shows mixed osteoblastic/osteolytic lesions, but in most cases of OS, the tumor is osteolytic (Kansara et al., 2014; Mutsaers and Walkley, 2014). OC as the only bone resorptive cells in the body, may be involved in the osteolytic process of OS. At present, it is believed that there are three possible models for bone degradation in OS. The first is in the vicious cycle model of tumor cells and OC, in which a series of growth factors and cytokines produced by tumor cells, directly and indirectly, stimulate OC differentiation and maturation, including M-CSF and RANKL, which are key factors for OC differentiation and maturation. Differentiated and mature OC further stimulate OS growth by releasing growth factors and minerals during bone resorption, such as insulin-like growth factor 1 (IGF1) or transforming growth factor β (TGF-β) (Alfranca et al., 2015; Lamora et al., 2016) (Figure 2; Table 1). The second is a model of bone degradation mediated by tumor cells, which are responsible for bone resorption by expressing collagenases including cathepsin K and MMPs. The third model is that tumor cells will occupy the eroded bone surface area and promote bone degradation through the expression of collagenolytic enzymes (Norregaard et al., 2021). In one study, it was confirmed that inoculation of OS cells with lytic potential into the femurs of OC-deficient mice not only did no tumor-induced bone destruction be observed, but tumor size was greatly reduced compared with OC-sufficient host mice (Clohisy and Ramnaraine, 1998). Therefore, OC may promote the growth of OS, targeted OC therapy can help prevent and treat bone destruction caused by OS. A literature report that zoledronate, an OC bone resorption inhibitor, can inhibit cell growth, induce cell apoptosis and reduce metastasis in OS seems to prove our conjecture (Lu et al., 2020a).

It is generally accepted that OS can regulate the generation of OC. However, there are conflicting results on the role of OS in OC formation and activity. Co-culture of human OS cell line MG63 with human peripheral blood monocytes revealed an increased number of OC (identified as TRACP-positive multinucleated cells) and increased absorptive activity (Costa-Rodrigues et al., 2011). Studies in mice inoculated with OS *in situ* have shown an increased number of OC in bone after OS inoculation compared with that without OS (Ohba et al., 2014). These results suggest that OS can regulate OC differentiation and maturation. Yet, as assessed by TRAcP 5 mRNA levels and immunohistochemistry, the number of OC was reduced in biopsies from OS patients compared with healthy controls. In addition, the reduction in OC number was more pronounced in patients with metastatic disease. A reduction in the number of OC was observed in mice transplanted with OS cells within the femur compared with PBS-treated mice. The reduction was more significant in mice transplanted with a metastatic OS cell line compared with a non-metastatic OS cell line (Endo-Munoz et al., 2010). It can be seen from this that broken bone cells may be related to OS distant metastasis negative correlation, a theory is in the initial stages of the disease, OS promote mature OC differentiation, differentiation of mature OC release a lot of growth factor in bone matrix to promote the growth of OS, and OS might get enough “nutrient” without distant metastases. OC may be used as diagnostic markers of OS metastasis.

A recent study also reported that the reduced number of OC in OS bone biopsies may be associated with chemotherapy efficacy. OS cells may have better chemotherapy efficacy when OC are differentiated and mature and retained in the bone microenvironment around OS cells. Advanced malignant tumors with chemo-resistant properties may contribute to the inhibition of OC generation, but the underlying mechanism needs further research to determine (Araki et al., 2021). Studies on OC and OS resistance may improve chemotherapy for primary or recurrent OS.

OC formation is dependent on RANK-RANKL signaling; therefore, loss of RANK expression in bone marrow lineage cells results in a lack of POC and mature OC (Ikebuchi et al., 2018; McDonald et al., 2021). Activation of the RANKL-RANK pathway in OS cell lines did not alter OS cell proliferation or migration, nor did it alter tumor growth *in vivo* (Navet et al., 2018). However, the use of RANK-Fc to inhibit OC cell lines effectively reduces the occurrence and metastasis of OS and improves the survival rate (Chen et al., 2015). It can be seen that RANKL-RANK pathway activation does not seem to be directly related to OS, and we speculate that it may promote the progression of OS by promoting OC differentiation and maturation. Whereas, some studies have found that RANK-containing exosomes secreted by OC can act on RANKL expressed on OS cells, suggesting that there may be a reverse signal transmission of RANK-RANKL between OS cells and OC (Garimella et al., 2014; Branstetter et al., 2015) (Figure 2; Table 1). Dinorumab is a monoclonal antibody against RANKL, which effectively inhibits the development and activity of OC (Reid and Billington, 2022). The combination of dinonumab with current chemotherapy regimens for OS may improve pathological fractures in OS.

Based on this, it can be hypothesized that bone remodeling in the OS microenvironment is related to the vicious cycle between OS and OC. On the one side, OC are the only bone-resorptive cells in the body. The other is RANK-RANKL signaling between OS cells and OC (Figure 2). However, with the deepening of research on exosomes, cytokines such as IL-1, PTHrP and non-coding RNA carried by exosomes secreted by OS cells and OC may play a direct role in this process (Leong, 2018). POC is considered as regulatory cells of the OC lineage, which can regulate bone and H-type angiogenesis, and may secrete cytokines in the bone microenvironment to promote OS growth (Peng et al., 2020) (Figure 2). The growth of primary OS is generally accompanied by the formation of a large number of new blood vessels, but whether POC promotes angiogenesis to promote OS is still unclear (Figure 2). Therefore, the specific mechanism of action of OC cell lines on OS needs to be further investigated.

## 3 Osteoblasts and osteocytes

OS cells can produce a large amount of bone-like matrix adjacent to it, thus forming abnormal bone structures, such as Codman’s triangle and solar radiation phenomenon. This abnormal osteogenesis of OS suggests that OS cells are closely related to osteoblasts and osteocytes.

Osteoblasts are derived from mesenchymal stem cells (MSCs) in the bone marrow and are differentiated through BMP and Wnt/β-Catenin signaling pathways. The differentiation of MSCs into mature osteoblasts involves a complex series of proliferation and differentiation steps. RUNX2 is essential for the early step before MSCs differentiation into osteoblasts and the maintenance of osteoblast function, while Osterix (also known as SP7) is mainly involved in osteoblast differentiation downstream of RUNX2, allowing pre-osteoblasts to differentiate into functional mature osteoblasts (Nishimura et al., 2012). Upstream of these transcription factors, signal transduction cascades must be activated by cytokines or growth factors such as TGF-β1, fibroblast growth factor (FGF) or wingless-type MMTV integration site family members (WNT). Most of these cytokines or growth factors are associated with OS. For example, TGF-β1 plays a key role in the interaction between OS cells and their microenvironment (Chang et al., 2021). *In vivo* experiments, the use of FGF receptor inhibitors can significantly inhibit the lung metastasis of OS. Overexpression of SMAD7 reduces primary tumor growth by blocking TGF-β activity in OS to affect the relationship between tumor and non-tumor cells (Lamora et al., 2014). Osteoblasts can eventually differentiate into endosteum cells and osteocytes (Capulli et al., 2014). Osteoblasts can promote osteogenesis through the deposition of organic matrix and its mineralization and are also able to influence OC formation with paracrine M-CSF and RANKL which OS can also secrete, and the aforementioned OC may mediate the malignant progression of OS (Chen et al., 2018). If OS originates from osteoblasts, OS-specific targets may be identified from osteoblasts.

Osteocytes are the most numerous among all bone cells and may play an important role in OS by coordinating the activities of OC and osteoblasts to maintain bone homeostasis (Buenzli and Sims, 2015). Osteocytes are also not just static mechanosensory cells and can contribute to bone remodeling by regulating bone formation and resorption. However, their roles in cancer invasion and metastasis are mostly unclear and underestimated. It is currently believed that osteocytes can directly participate in the formation of osteolytic and osteoblastic lesions by regulating OC and osteoblasts, respectively. In breast cancer, osteocytes can promote the proliferation and migration of breast cancer cells through the potential CXCL1/2 mechanism (Dwivedi et al., 2021). Prostate cancer can promote the vicious cycle of bone metastasis progression by inducing osteocytes to secrete GDF15 that stimulates prostate cancer growth and invasion (Wang et al., 2019). In addition, osteocytes may promote multiple myeloma tumor cell proliferation and bone destruction through the Notch signaling pathway (Delgado-Calle et al., 2016). However, the regulatory role of osteocytes in OS remains to be further discovered.

Whether osteoblasts and osteocytes have the mechanism of regulating the growth and metastasis of OS needs to be further studied. Whether OS originates from osteocytes, osteoblasts or MSCs remains to be determined, but the growth of primary OS cannot be separated from the bone, and a large part of the bone is composed of osteoblasts and osteocytes (Lin et al., 2017). So the occurrence and evolution of OS may be related to osteoblasts and osteocytes.

## 4 Mesenchymal stem cells

Mesenchymal stem cells (MSCs) in the bone microenvironment, as the most important influencing factor in the bone microenvironment, play an important role in the growth, progression, metastasis, drug resistance and targeted therapy of OS (Corre et al., 2020).

**FIGURE 3:**
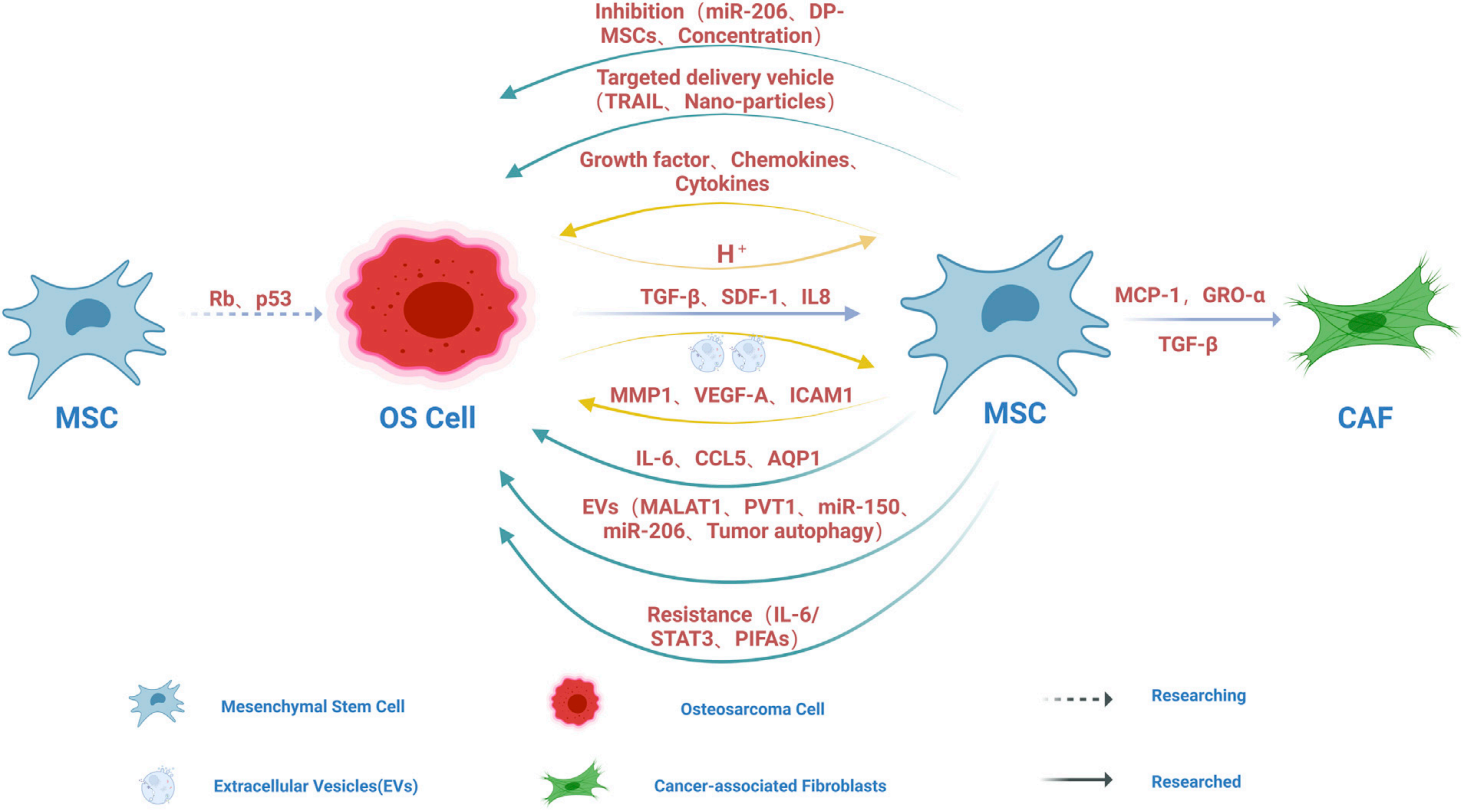
Schematic diagram of the cell cross-talk between OS and MSC. The inactivation of tumor suppressor genes such as Rb and P53 may lead to the mutation of MSCs to OS. OS can induce the migration of MSCs into OS by TGF-β and SDF1, and can induce the differentiation of MSCs into CAF by CP-1, GRO-α and TGF-β. Exosomes (EVs) secreted by OS promote the release of MMP1, VEGF-A and ICAM1 from MSCs by interacting with MSCs, which supports that MSCs can promote the local invasion and distant metastasis of OS by participating in bone remodeling and angiogenesis. OS can secrete IL-8 and trigger the expression of IL-8 in MSCs to promote the growth and metastasis of OS. Hypoxic conditions in OS microenvironment lead to hypoxic glycolysis in OS, which leads to extracellular matrix acidification, which in turn helps activate MSCs to release a large number of growth promoting factors, chemotaxis and cytokine secretion that affect OS behavior. MSCs can secrete a series of factors that directly promote OS growth and metastasis, such as IL-6 and CCL5. BM-MSCs can promote OS metastasis and invasion by upregulating AQP1 level. EVs secreted by MSCs can directly promote the growth and metastasis of OS, such as MALAT1, PVT1, miR-150, miR-206, and can also promote the growth and metastasis of OS by promoting oncogenic autophagy in OS. MSCs can participate in the resistance of OS to anticancer drugs through IL-6/STAT3 and PIFAs. MSCs can be used as an effective platform for targeted delivery of therapeutic nanomedicines, alone or in combination with other OS therapies, such as delivery of TRAIL and nanoparticles. BM-MSC-derived EVs containing miR-206 could inhibit OS growth by targeting TRA2B. Compared with BM-MSCs, DP-MSCs showed anti-tumor effect. Low concentrations of Ad-Mscs can inhibit tumor growth, while higher concentrations can stimulate tumor growth. The arrow represents the direction, the dotted line represents the unproven, and the solid line represents the proven.

MSCs may be the precursor cells of OS. It has been found that the inactivation of some important tumor suppressor genes, such as Rb and P53, may lead to the transition of MSCs to OS (Rubio et al., 2013; Sarhadi et al., 2021) (Figure 3). Further studies found that these OS-derived MSCs had stronger osteogenic differentiation ability and could promote local invasion and lung metastasis of OS. Regretfully, MSCs did not have chromosomal rearrangements compared with normal MSCs and did not induce tumors in immunodeficient mice. Based on this view that OS originate from MSCs, a deeper mechanistic study of OS deserves further exploration. This revealed that MSCs may play a non-negligible role in the origin of OS, which is worthy of further exploration.

OS can regulate the migration and differentiation of MSCs and secrete a large number of cytokines to promote the growth and metastasis of OS. MSCs can be induced to migrate to OS by transforming growth factor (TGF-β) and stromal-derived factor (SDF1), and MSCs can also be induced to differentiate into cancer-associated fibroblasts (CAF) by monocyte chemoattractant protein (MCP)-1, growth-regulated oncogene-α (GRO-α) and transforming growth factor (TGF)-β1 (Figure 3; Table 1). Cancer-associated fibroblasts which contribute to OS progression and metastasis are the key components of TME (Pietrovito et al., 2018). The differentiation of bone marrow-derived MSCs into CAFs is a multi-step and complex biological process that may involve epithelial-mesenchymal transition, bone marrow-derived progenitor cells, cell communication, and cytokines (Zhu et al., 2016). In addition, EVs secreted by OS cells interact with MSCs to promote the release of matrix metalloproteinase-1 (MMP1), angiogenic factor-A (VEGF-A) and intercellular adhesion molecule-1(ICAM-1) from MSCs, which supports that MSCs can promote local invasion and distant metastasis of OS by participating in bone remodeling and angiogenesis (Figure 3). OS can secrete interleukin-8 (IL-8) to trigger the expression of IL-8 in MSCs, and MSC-derived IL-8 promotes OS cell growth and metastasis through C-X-C chemokine receptor-1 (CXCR-1)/Akt signaling (Du et al., 2018) (Figure 3; Table 1). When adipose-derived MSCs (AD-MSCs) were treated with extracellular vesicles from OS, MSCs increased the expression of angiogenic factor (VEGF) which can promote neovascularization in the bone microenvironment, further enhancing tumor growth and metastasis (Mannerstrom et al., 2019) (Table 1). It has been shown that MSCs expressing hypoxia Inducible Factor-1α (HIF-1α) produce extracellular vesicles that can activate Notch signaling and promote matrigel angiogenesis in mice *in vitro* and *in vivo* (Gonzalez-King et al., 2017). Hypoxic conditions in the tumor microenvironment may lead to hypoxic glycolysis of OS, which leads to acidification of the extracellular matrix, which in turn helps to activate MSCs to release a large number of factors that affect the behavior of OS, such as growth factors (colony stimulating factor 2 (CSF2)/granulocyte-macrophage colony-stimulating factor (GM-CSF), CSF3/granulocyte colony-stimulating factor (G-CSF) and bone morphogenetic protein 2 (BMP2)), chemokines (C-C chemokine ligand 5 (CCL5), C-X-C Motif Chemokine Ligand 5 (CXCL5) and CXCL1 (GRO-α)), and cytokines (IL-6 and IL-8), while increasing the expression of CXCR4 (Avnet et al., 2017) (Table 1).

MSCs can secrete a series of factors that directly promote OS growth and metastasis, such as IL-6 and CCL5 (Xu et al., 2009) (Figure 3). Tumor-associated MSCs in the bone microenvironment activate the inflammatory NF-kB signaling cascade and induce the secretion of the cytokine CCL5, which contributes to OS migration and metastasis (Wang et al., 2015) (Table 1). Bone marrow MSC (BM-MSC) conditioned medium has been reported to increase aquaporin 1 (AQP1) expression levels in OS, and it has been demonstrated that TME BM-MSCs can promote metastasis and invasion by upregulating AQP1 levels (Pelagalli et al., 2016) (Figure 3).

Extracellular vesicles secreted by MSCs can promote OS growth and metastasis. In a recent study, Li et al. (2021) showed that bone marrow MSC (BM-MSC) -derived extracellular vesicles (EVs) promote the proliferation, invasion and migration of OS via the lncRNA MALAT1/miR-143/NRSN2/Wnt/β-Catenin axis (Figure 3; Table 1). BM-MSC EV carried MALAT1 into OS, increased the expression of MALAT1 and NRSN2, decreased the expression of miR-143, and activated the Wnt/β-catenin pathway in OS. *In vivo* experiments confirmed that BMSC-EV promoted tumor growth in nude mice (Li et al., 2021). BM-MSC-derived exosomes promote the growth and metastasis of OS through PVT1/ERG pathway (Zhao et al., 2019) (Figure 3). MSCs-derived exosomes carrying miR-150 inhibit the proliferation and migration of OS cells by targeting IGF2BP1 (Xu et al., 2020) (Figure 3). Another mechanism by which BMSC-EV promotes tumorigenesis and metastasis is by promoting oncogenic autophagy in OS (Huang et al., 2020) (Figure 3).

The presence of MSCs in the bone microenvironment is an important component of OS cell resistance to anticancer drugs (Birru et al., 2020). OS increases IL-6 expression in MSCs, which in turn activates STAT3 signaling in OS, which promotes OS cell survival by protecting OS from drug-induced apoptosis (Figure 3; Table 1). Low expression of STAT3 in OS patients can reduce the recurrence after surgery and chemotherapy (Tu et al., 2016). Platinum-based chemotherapeutic agents are classic agents for OS treatment. During treatment with platinum analogues, endogenous MSCs have been reported to be activated and release platinum-induced polyunsaturated fatty acids (PIFAs), 12-oxo-5, 8, 10-hexadecanoate (KHT), and hexadecane-4, 7, 10, 13-tetraenoic acids (16: 4 (n-3)), these PIFAs can protect OS from a range of chemotherapeutic agents (Roodhart et al., 2011) (Figure 3). Interestingly, blocking the central enzymes involved in the production of these PIFA (cyclooxygenase-1 and thromboxane synthase) prevented MSC-induced resistance.

Targeted therapy is attracting more and more attention as a new option for cancer treatment. The unique ability of MSCs to homing and transplant in the tumor stroma makes them effective targeted delivery vectors to carry therapeutic agents to the tumor stroma. For example, TNF-related apoptosis-inducing ligand (TRAIL) delivered by adipose-derived MSCs (AD-MSCs) has anti-tumor effect on OS, and TRAIL delivered by Ad-MSCs can effectively kill OS (Figure 3). Because MSCs have a longer half-life, they can stably deliver TRAIL and secrete co-factors (Gamie et al., 2017). MSCs can also serve as nanoparticle delivery vehicles, and MSCs-loaded photosensitizer-containing nanoparticles have been shown to trigger OS cell death *in vitro* upon specific photoactivation via the release of reactive oxygen species (ROS) (Figure 3). Unfortunately, anti-tumor drugs used in MSC delivery systems may kill MSCs, leading to treatment failure (Duchi et al., 2013). MSCs can deliver functional photosensitizer-modified nanoparticles *in vitro* and *in vivo* and inhibit OS tumor growth (Lenna et al., 2020). MSCs may serve as an effective platform for the targeted delivery of therapeutic nano-medicines, alone or in combination with other OS treatment modalities. Mesenchymal stem cell-derived exosomes have been used as nano-drug carriers of doxorubicin to target OS therapy through the SDF1-CXCR4 axis (Wei et al., 2022) (Table 1).

MSCs is a double-edged sword of OS. Although many studies have identified the growth-promoting role of MSCs in OS, a few studies have also demonstrated that MSCs can effectively alleviate and inhibit the recurrence, proliferation and metastasis of OS. BM-MSC-derived EVs containing miR-206 have been reported to inhibit OS growth by targeting TRA2B (Zhang et al., 2020) (Figure 3; Table 1). It was found that MSCs did not promote local recurrence or post-recurrence tumor size in OS, but intravenous administration of MSCs did accelerate lung metastasis (Aanstoos et al., 2016). Compared with bone marrow-derived MSCs (BM-MSCs), dental pulp-MSCs (DP-MSCs) have more anti-tumor effects and form dentin-pulp-like complexes that are resistant to tumor transformation (Shen et al., 2019) (Figure 3). Studies have shown that local injection of different concentrations of adipose-derived MSCs (AD-MSCs) into the tumor site will lead to different effects. Low concentrations of AD-MSCs have an inhibitory effect on cancer, while higher concentrations can stimulate tumor growth (Lee et al., 2015) (Figure 3). Thus, the effect of MSCs on OS seems paradoxical, depending on the source of MSCs, the tumor site, and the content of signaling molecules in the tumor microenvironment (Eiro et al., 2021).

At present, the application of MSCs mainly focuses on two aspects. One is to use MSCs themselves to achieve the activation or inhibition of target signaling pathways and the secretion of related cytokines through secretion regulation to limit tumor growth. On the other hand, they are used as carriers to achieve targeted therapy at tumor sites (Figure 3). The extracellular vesicles secreted by them, especially exosomes, have broader development prospects as molecular drugs or gene carriers. Current studies have mainly focused on the effect of BM-MSCs on malignant lesions of OS. There are few relevant studies on MSCs derived from other tissues, such as adipose-derived MSCs (AD-MSCs), dental pulp-derived MSCs (DP-MSCs) and human umbilical cord (HUC-MSCs) or embryonic stem cells. There have been no functional comparisons between MSCs derived from different tissues. In addition, the mechanisms underlying the interaction between MSCs and OS need to be further investigated, and the mechanisms underlying their effects may include induction of differentiation, immune regulation, cell fusion, and paracrine effects. A more in-depth study of this interaction will likely greatly aid in the search for new drug targets and treatments for OS.

## 5 Fibroblasts

Originally defined as cells that reside in connective tissue and synthesize collagen, fibroblasts are currently thought to be derived from interstitial cells of the mesenchymal lineage (Chen et al., 2021). Due to the substantial phenotypic and functional heterogeneity of fibroblasts, the exact cellular origin and function of these cells remain obscure and difficult to determine (Kalluri, 2016a). Fibroblasts are multifunctional cells that are seen in tissue injury, during wound healing, and in tumor formation (Driskell et al., 2013; Arina et al., 2016). A population of fibroblasts found in primary and metastatic tumors collectively referred to as cancer-associated fibroblasts (CAFs) (Sahai et al., 2020). CAFs are a kind of stromal cell population with similar cell of origin, phenotypic and functional heterogeneity, which is an important component of TME. Through a variety of pathways, activated CAFs secrete growth factors, inflammatory ligands and extracellular matrix proteins, which can promote tumor growth, angiogenesis, invasion and metastasis, extracellular matrix (ECM) remodeling and even drug resistance (Chen and Song, 2019; Mao et al., 2021).

Previous studies have shown that α-smooth muscle actin (α-SMA), fibroblast activation protein (FAP), S100A4 and platelet-derived growth factor receptor β (PDGFRβ) can be used as markers to define CAFs (Kalluri, 2016b). Nevertheless, none of these cell surface markers was exclusively expressed by CAFs, which also highlights the heterogeneity of fibroblasts. In TME, CAFs can regulate tumor progression and immunity by producing growth factors, cytokines, and chemokines, including CCL2, CCL5, CSF1, CXCL5, CXCL9, CXCL10, and CCL5. CXCL12 (also known as stromal cell-derived factor 1 (SDF1)), HGF, IGF1, Connective tissue growth factor (CTGF), platelet-derived growth factor (PDGF), Vascular endothelial growth factor (VEGF), IL-1, IL-4, IL-6, IL-8, IL-10, leukemia inhibitory factor (LIF), prostaglandin E2 (PGE2) and TGF-β (Chen et al., 2021). Among them, CCL5, CSF1, CXCL5, CXCL9, CXCL10, CXCL12, HGF, IGF1, CTGF, PDGF, VEGF, IL-1, IL-4, IL-6, IL-8, IL-10, LIF, PGE2, TGFβ are closely related to the invasion and metastasis of OS (Lamora et al., 2016; Dang et al., 2017; Sun et al., 2017; Chellini et al., 2018; Gross et al., 2018; Li et al., 2018; Raucci et al., 2019; Sergi et al., 2019; Lu et al., 2020b; Deng et al., 2020; Niu et al., 2020; Smeester et al., 2020; Wen et al., 2020; Xing et al., 2020; Yu et al., 2020; Ren et al., 2021; Sun et al., 2021; Yati et al., 2022) (Table 1). Recent studies in pancreatic cancer suggest that CAFs may also have a tumor suppressor function (Biffi and Tuveson, 2021).

CAFs in TME play an important role in regulating the antitumor activity of tumor-infiltrating immune cells, including innate and adaptive immune cells (Mao et al., 2021). In addition, they promote the expression of immune checkpoint molecules and ECM remodeling, indirectly affecting the recruitment and activity of immune cells. Through the secretion of cytokines, chemokines, and other effector molecules, including TGF-β, CXCL2, collagen, MMP, and laminin, CAFs can promote immune cells to participate in the occurrence and development of cancer, while promoting the degradation and remodeling of ECM (Ziani et al., 2018). Many studies have shown that the interaction between CAFs and immune cells and other immune components can regulate the tumor immune microenvironment (TIME), thereby inhibiting anti-tumor immune response.

CAFs have been traditionally identified as tumor-promoting components. Based on this, we hypothesized that CAFs may promote the malignant progression of OS through the production of growth factors, cytokines and chemokines and immunosuppression in TME. Targeted CAFs may be one of the treatment options for OS.

## 6 Vascular endothelial cells and pericytes

Vascular endothelium Endothelial cells (EC) are multifunctional structures that separate circulating blood from tissues. Moreover, in addition to regulating and maintaining blood fluidity, it can deliver water and nutrients, maintain metabolic homeostasis, transport immune cells, activate innate and acquired immune responses, and generate blood vessels (Sobierajska et al., 2020). Like other organs, OS also requires a blood supply to provide nutrients and oxygen for growth and to remove metabolic wastes (Lugano et al., 2020). Tumors satisfy their vascular supply through angiogenesis. Tumors regulate their microenvironment by releasing many cytokines, chemokines, and growth factors to activate normal, quiescent endothelial cells and adapt them to angiogenesis. Endothelial cells may undergo an endothelial-to-mesenchymal transition to become CAFs. It has been proven that SDF-1 in CAFs recruits EC that promote angiogenesis, and the induction of IL-8 secretion by CAFs isolated from patients with metastatic colon cancer also promotes neovascularization (Sobierajska et al., 2020). But on miR-126, tumor angiogenesis and cell proliferation seem to be tangled. MiR-126 is an endothelium-specific miRNA that acts as a negative regulator of VEGF-A to regulate angiogenesis signaling and vascular integrity. Overexpression of miR-126 in endothelial cells has been observed to enhance VEGF-A activity and promote angiogenesis by inhibiting the expression of Sprouty-associated protein-1 (Spred-1) (Wang et al., 2008) (Table 1). However, in the early invasive stage of oral squamous cell carcinoma and cervical cancer, low miR-126 expression promoted tumor progression by promoting angiogenesis (Sasahira et al., 2012; Huang and Chu, 2013). OS often occurs in distant hematogenous metastasis. Tumor metastasis first decomposes the basement membrane, invents the matrix, and infiltrates into the blood circulation. Among them, tumor cell intravasation is the rate-limiting step of metastasis, which can regulate the number of circulating tumor cells, and the trans-endothelial migration (TEM) of tumor cells is the key part of intravasation (Wan et al., 2013). This fraction can be divided into migration between two endothelial cells and migration through a single endothelial cell. However, this barrier can be regulated by factors present in the tumor microenvironment through endothelial cells constitute a barrier to tumor cell intravasation (Zervantonakis et al., 2012). During migration, the interaction between tumor cells and EC induces contraction and disruption of endothelial cell-cell contacts and secretion of proinflammatory factors by the latter. The blood vessels generated during tumor progression are usually immature and do not have proper junctional contacts between EC, which can allow tumor cells to intravasate through the blood barrier for distant metastasis (van Zijl et al., 2011).

Pericytes are mesenchymal cells that tightly encase small blood vessels, and their role is to interact with EC, which are believed to promote angiogenesis under physiological conditions (Lindblom et al., 2003; Geevarghese and Herman, 2014). Endothelial cells can recruit pericytes through PDGF-B/PDGFR-β signaling (Table 1). In addition to PDGFR-β, multiple signaling pathways allow communication between pericytes and endothelial cells, including angiopoietin I (Ang I), which regulates endothelial cell viability and TGF-β, which regulates pericyte differentiation (Chang et al., 2015). Pericytes have recently received attention as important mediators of cancer vascular biology and angiogenesis. For example, in melanoma, FAK in pericytes negatively regulates Gas6/Axl signaling to inhibit tumor angiogenesis and tumor growth (Lechertier et al., 2020) (Table 1). Loss of pericytes in tumor-associated vessels increases vascular permeability and reduces vascular integrity, thereby promoting tumor metastasis (Gerhardt and Semb, 2008). The presence and distribution of microvascular pericytes have been detected in OS specimens, but pericyte coverage in specimens has not been significantly correlated with tumor growth or metastasis (Hemingway et al., 2012).

Recently, progress has been made in the development and application of targeted anti-angiogenic drugs. Targeted anti-angiogenesis therapy includes monoclonal antibodies against VEGF (bevacizumab), tyrosine kinase inhibitors (sorafenib, apatinib, pazopanib, and regofenib), and human recombinant endostatin (Endostar) (Liu et al., 2021). However, alternative targeted anti-angiogenesis regimens are still in their infancy and face numerous problems before they can be widely used in the clinic. For example, how can we predict the efficacy of anti-angiogenic targeted therapies for OS, and which new drugs will be most effective in combination with traditional therapies? More detailed clinical studies are needed to establish reasonable norms and guidelines for the application of these reasonable treatment alternatives. With the development of technology and extensive research, targeted anti-angiogenesis therapy may become a powerful weapon for us to effectively manage patients with OS.

## 7 Macrophages

Macrophages are the first immune cells during embryonic development and are involved in organ development, homeostasis, immunity and repair *in vivo*. Macrophages are involved in bone homeostasis and immunity in the bone microenvironment and have central functions in bone immunology (Tsukasaki and Takayanagi, 2019). Three known distinct macrophage populations have been identified in bone tissue: the macrophage population of bone marrow macrophages, OC, and bone macrophages (Cersosimo et al., 2020). Our current understanding of macrophages has evolved from being considered simple phagocytes to grasping the regulatory factors involved in the management of a myriad of cellular processes (Cox et al., 2021). A major influencing component of the tumor microenvironment is tumor-associated macrophages (TAMs), which are immune cells involved in the inflammatory response and tissue homeostasis (Murray and Wynn, 2011). Increased TAMs infiltration has been consistently associated with poor patient outcomes in most tumors, highlighting their value as potential diagnostic and prognostic biomarkers in tumor tissues (Cersosimo et al., 2020).

TAMs are an important component of tumor stroma and are closely involved in many stages of tumor growth. In several cases, macrophages can account for up to 50% of the tumor mass, and their abundance is associated with poor clinical outcomes. A large amount of evidence has shown that TAMs promote tumor growth by promoting angiogenesis, immunosuppression and chronic inflammation, and can also affect tumor resistance after conventional anti-tumor therapy (Mantovani et al., 2002). At present, TAMs are believed to play three different roles in promoting tumor growth and metastasis: first; TAMs promote tumor cell invasion into the vasculature through MCSF-1 from tumor cells and epidermal growth factor (EGF) from macrophages and their receptors, thereby promoting tumor spread (Condeelis and Pollard, 2006). Second, TAMs promote tumor growth by inhibiting adaptive and innate antitumor immunity by secreting immunosuppressive molecules, including TGFβ, IL10, arginase-1 (Arg-1) and NO (Cersosimo et al., 2020). Third, TAMs have proangiogenic properties, thereby promoting tumor growth and recurrence. Notably, macrophages expressing VEGF-A and tyrosine kinase with immunoglobulin and epidermal growth factor homology-2 (Tie2) have been found to play a crucial role in the recovery of tumor vasculature and tumor recurrence after doxorubicin treatment (Zhang et al., 2019). The higher density of M2-type TAMs found in lung metastases compared to primary OS may be related to increased tumor invasiveness caused by proinflammatory molecules (Han et al., 2019).

Macrophages can also secrete a series of cytokines to promote the proliferation and metastasis of OS. CCL18 is a chemokine released by M2 macrophages, and CCL18 expression correlates with the proliferation and invasion of OS in OS tissues. In addition, the number of CCL18^+^ TAMs identified was higher in metastatic OS tissues compared to primary OS. Studies using a xenograft model showed that CCL18 increased tumor size and induced lung metastasis, suggesting that TAMs can promote OS growth and distant metastasis by secreting CCL18 (Su et al., 2019) (Table 1). Inhibition of cyclooxygenase-2 (COX2) reduced the migration ability of OS, and it was found that COX2 overexpression in OS co-cultured with TAMs increased the expression level of p-STAT3, thereby promoting the metastasis of OS (Han et al., 2019). All-trans retinoic acid (ATRA) treatment can inhibit OS metastasis by preventing M2 polarization and TAM-induced MMP12 secretion (Zhou et al., 2017a).

Unexpectedly, in a cohort study, patients with advanced OS with high tumor-associated macrophage infiltration had longer disease-free survival and fewer distant metastases. This may be because macrophages have high plasticity and can acquire opposite phenotypes: inflammatory phenotype (M1) and anti-inflammatory phenotype (M2) (Sica and Mantovani, 2012). It is believed that OS metastasis can be regulated by changing the ratio of M1 to M2 macrophages, such as switching macrophage polarization to the TAMs-like intermediate M1/M2 phenotype, which can inhibit OS proliferation (Cersosimo et al., 2020). In preclinical models of OS, M2-type TAMs is associated with OS progression, angiogenesis, and metastasis (Dumars et al., 2016). Inhibition of M2 macrophage differentiation in tumor-associated macrophages can produce anti-tumor and anti-metastatic effects (Kimura and Sumiyoshi, 2015). Zoledronate, a nitrogen-containing bisphosphonate, significantly reduced OS-induced *in vivo* lung metastasis and also modulated TAMs polarization of the M2 to M1 phenotype (Cersosimo et al., 2020).

Like many solid tumors, macrophages are the main immune components in the OS microenvironment, and therapies focusing on targeting TAMs have become a hot topic of immunotherapy. Current macrophage-centered therapies include the elimination of TAMs and repolarization of TAMs into pro-inflammatory M1 macrophages. Extracellular vesicles or exosomes containing cytokines that promote OS growth, invasion, and metastasis and genetically engineered macrophages may be the future direction.

## 8 Lymphocytes

As recognized, lymphocytes are active elements of the tumor microenvironment and participate in the growth and metastasis of OS. Lymphocytes include natural killer cells (NK cells), T lymphocytes, and B lymphocytes.

NK cells attack tumor cells and release tumor antigens and risk-associated molecular patterns (DAMPs), which initiate and perpetuate immune responses by stimulating professional antigen-presenting cells (APCs) (Lettieri et al., 2016). CD8 cytotoxic T lymphocytes (CTLs) are the main effector cells of the adaptive immune response and are activated or clonally proliferated by dual signals before killing tumor cells. Upon receiving signals from major histocompatibility complex (MHC) class I antigen peptide molecules, CD4^+^T cells also release cytokines such as IL-2 and IFN-γ on the surface of licensed professional APCs, which play an important role in regulating antitumor effects (Haworth et al., 2015). The results of one study showed that OS infiltration of CD4^+^T cells and CD8^+^T cells was associated with OS patient survival. CD4^+^T cells may improve the prognosis of OS, and CD8^+^T cells may improve the overall survival and progression-free survival (PFS) of OS patients (Casanova et al., 2021). TLR4 inhibits the progression of OS lung metastasis in a mouse model by increasing CD8^+^T cell infiltration (Yahiro et al., 2020). It has been suggested that the absence or weak infiltration of CD4^+^ and CD8^+^T cells is one of the possible explanations for the aggressiveness of OS (Alves et al., 2019).

The location and density of B cells in the TME vary across cancer types. Tumor-infiltrating B cells (TIL-B) reside primarily in trtiary lymphoid structures (TLS), which are ectopic lymphopoiesis that can develop in the TME. Most are in the TLS of the germinal center, where they undergo a full maturation process from naive B cells to memory B cells and plasma cells (PCs), which propagate into the tumor. B cells can present antigens to T cells either directly or via immune complexes endocytosed by dendritic cells. This amplification circuit is particularly effective in less immunogenic tumors that are unable to directly activate T cells. Antibodies produced by PCs can also promote the anti-tumor effector functions of macrophages and NK cells. In contrast, in immature TLS tumors lacking germinal centers, B cells adopt a regulatory phenotype and suppress the immune response. Immune complexes may also activate complement or macrophages to contribute to pro-tumor inflammation. Thus, the role of B cells is complex, depending on the nature of the antigen they recognize and the composition of the TME (Engelhard et al., 2021; Laumont et al., 2022). Thus, the density of B cells and mature TLS is a major predictor of response to immunotherapy, which allowed us to extend it to OS with poor immunogenicity.

The immune microenvironment is a hot topic at present. Exploring the multi-factor prediction model, diagnostic model and grading scores of OS with immune component is to promote precision treatment. Try to combine immunotherapy with other therapies by developing new approaches that are more effective than existing therapies under the premise of ensuring safety. The immune function between NK cells, T lymphocytes and B lymphocytes and OS needs to be further studied. How immune suppression or evasion processes occur and what mediators serve as their cross-talk may provide new insights into the development of new targets for OS immunotherapy.

## 9 Physical and chemical properties of bone microenvironment

The changes in physicochemical properties in the tumor microenvironment, such as hypoxia and acidity, are also closely related to the occurrence, development and metastasis of tumors. OS is no exception. Hypoxia is mainly involved in the regulation of OS by activating HIF (Zhang et al., 2021). For example, HIF-1α can promote distant metastasis of OS under hypoxia (Guan et al., 2015). In addition, non-coding RNA, such as miR-20b and miR-33b, also act on HIF-1α to regulate the proliferation and invasion of OS under hypoxia (Liu et al., 2016; Zhou et al., 2017b). LncRNA MALAT1 can also promote angiogenesis in OS cells under hypoxia, thereby promoting distant metastasis of OS (Zhang et al., 2017). Therefore, targeting HIF activated in OS hypoxic environment may bring new benefits to OS patients.

The PH stability of the bone microenvironment is inseparable from bone homeostasis. The dissolution of bone matrix in an acidic environment creates potential conditions for local invasion and distant metastasis of OS. In addition, the extracellular acidic environment can activate MSCs through the NF-κB inflammatory signaling pathway, and MSCs can promote the progression of OS and regulate the chemotherapy resistance of OS by releasing a variety of cytokines, such as IL6, IL8, and CCL5, in a paracrine manner. At the same time, the expression of several cytokines, such as CSF3, IL-1A, IL-23A, IL-1RN, CXCL, CCR7, CSF2/GM-CSF, CSF3/G-CSF, and MMP-2, was increased in the OS microenvironment under acidic conditions (Yang et al., 2020). Therefore, various cytokines secreted by the acid-mediated microenvironment play an important role in the progression of OS, and the intervention of the acidic environment provides a possible therapeutic strategy for OS in the future.

## 10 Conclusion

In the past decade, a large body of evidence supports that the bone microenvironment, which is composed of OC, osteoblasts, osteocytes, MSCs, fibroblasts, endothelial cells, pericytes, macrophages, and immune cells, promotes tumor progression and metastatic spread of OS. It is found that the cross talk in the bone microenvironment plays a very important role in the malignant progression of OS. Therefore, the communication mediators mediating cross-talk in the bone microenvironment are particularly important, such as various chemokines, inflammatory factors, growth factors, extracellular vesicles, and exosomes. The heterogeneity of OS leads to the lack of specific anti-OS targets. Targeting other cells and intercellular communication mediators in the bone microenvironment, targeted nanomedicine and targeting carrier will be a direction for the treatment of OS in the future.
